# Regulation of pseurotin A biosynthesis by GliZ and zinc in *Aspergillus fumigatus*

**DOI:** 10.1038/s41598-023-29753-z

**Published:** 2023-02-10

**Authors:** Hyewon Seo, Suzie Kang, Dongho Lee, Cheol-Won Yun

**Affiliations:** 1 School of Life Sciences and Biotechnology, Korea University, Anam-Dong, Sungbuk-Gu, Seoul, Republic of Korea; 2 Department of Plant Biotechnology, College of Life Sciences and Biotechnology, Korea University, Seoul, Republic of Korea; 3 NeuroEsgel Co., Anam-Dong, Sungbuk-Gu, Seoul, Republic of Korea

**Keywords:** Fungi, Microbial genetics, Pathogens

## Abstract

Recently, we reported that zinc regulates gliotoxin biosynthesis via ZafA, which is a zinc-responsive transcriptional activator. From an HPLC analysis of culture media of *Aspergillus fumigatus*, we found a trend of decreasing gliotoxin production but increasing pseurotin A and fumagillin production in proportion to the zinc concentration. The expression of the genes involved in pseurotin A biosynthesis was upregulated under high zinc concentrations. Furthermore, upregulated expression of pseurotin A biosynthetic genes and higher production of pseurotin A were observed in the *zafA* deletion strain. Interestingly, the deletion of *gliZ,* a transcriptional activator of gliotoxin biosynthesis genes, resulted in upregulated expression of pseurotin A biosynthetic genes and increased production of pseurotin A. We detected upregulation of *fumR* expression in the *gliZ* and *zafA* deletion mutants. The overexpression of *gliZ* observed in the *zafA* deletion mutant resulted in the failure of the mutant to increase pseurotin A production, which is a phenotype of the *zafA* deletion mutant. These results suggest that ZafA sequentially regulates pseurotin A biosynthesis through GliZ. Finally, we found through a murine virulence test that the *gliZ* and *fumR* double-deletion mutants showed a delayed death rate compared with the single-deletion mutants of either *gliZ* or *fumR*. Taken together, these results suggested that the biosynthesis of gliotoxin and pseurotin A are regulated in opposite ways by zinc utilization and that each secondary metabolite is synthesized when the synthesis of another secondary metabolite fails to protect it against the defense system of the host.

## Introduction

*Aspergillus fumigatus* is a representative opportunistic fungal pathogen and causes various lung diseases, such as asthma and cystic fibrosis^1,2^. The hyphae of *A. fumigatus* spread to the lung tissues and cause invasive aspergillosis (IA)^3^. Neutrophils are immune cells that immediately respond to microbial infection and are primarily responsible for the removal of microbial pathogens in animals^4–6^. Neutrophils can inhibit fungal growth in diverse ways, including zinc sequestration^7^. Zinc sequestration makes zinc unavailable to *A. fumigatus* by pumping out all zinc ions present in the phagosome^8–10^. When *A. fumigatus* is exposed to zinc sequestration, the fungi upregulate the expression of the transcription factor ZafA, which is part of a defense system of fungi^11,12^.

*Aspergillus fumigatus* is also constantly trying to overcome pathogen killing by neutrophils^13^. Many strategies by which fungi protect themselves from the host defense system have been reported, and the secretion of secondary metabolites is one mechanism^14^. Secondary metabolites are small molecules that are synthesized by diverse microbial species. Although they are not directly involved in life-sustaining activities, secondary metabolites have species-specific characteristics^15,16^ and are involved in competition with other organisms. For example, dihydroxynaphalene (DHN) melanin, fumagillin, and gliotoxin are secondary metabolites that protect against the host immune system^14,17–22^. Other types of metabolites that compete with other microbes in the environment are also present^14,23–33^.

Fungi commonly form gene clusters that are involved in the synthesis of certain secondary metabolites. The region of the fungal genome where genes are clustered is called the biosynthetic gene cluster (BGC). Each BGC contains central biosynthesis genes, which encode the enzymes responsible for biosynthesizing secondary metabolites, such as polyketide synthases (PKSs) and nonribosomal peptide synthetases (NRPSs)^34–37^. The transcription factors that regulate the expression of BGCs are categorized into two types. One is the “global transcriptional activator” type, which regulates the expression of multiple BGCs in response to environmental stimuli. LaeA is a global transcriptional activator that responds to light signals^38,39^. The other type of BGC transcription factor is the “pathway-specific transcription activator”, which is localized in a specific BGC and regulates the expression of other genes involved in the corresponding BGC. Most fungal BGCs contain their own pathway-specific transcription factors. GliZ is the gliotoxin BGC pathway-specific transcription factor that is responsible for the expression of other genes in that cluster^40^. FumR (FapR) is a pathway-specific factor that acts on both of the intertwined BGCs of fumagillin and pseurotin A^41^. In *A. flavus*, a pathway-specific factor, AflR, acts on the BGC of aflatoxin^42^.

To date, many studies on genetic mechanisms have shown the mechanisms involved in secondary metabolite expression, and some response factors to micronutrient stimuli have been reported. Additionally, ZafA has been reported to promote the expression of *gliZ* in the gliotoxin BGC^43,44^. The relationships between ZafA and the expression of secondary metabolites, including gliotoxin, pseurotin A and fumagillin, were investigated in this report, and we identified a novel interaction between gliotoxin and pseurotin A biosynthesis.

## Results

### High-zinc conditions increased pseurotin A and fumagillin production

**Figure 1:**
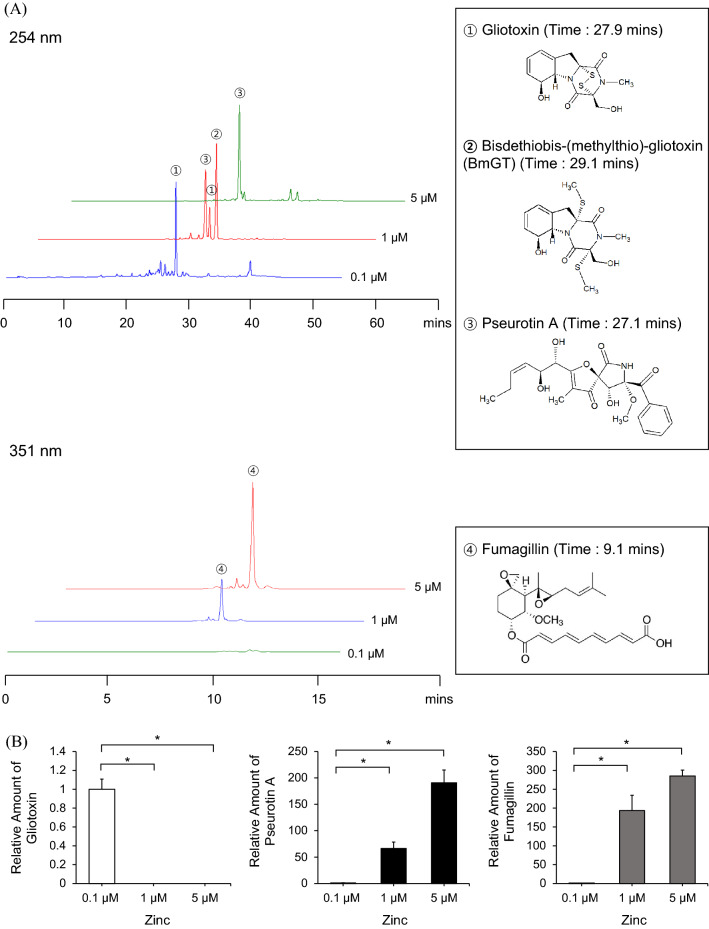
Biosynthesis of pseurotin A and fumagillin was increased by zinc. (**A**) The secondary metabolites were extracted from the culture media supernatant of the cells cultured at the indicated zinc concentrations and separated by HPLC. The peaks that appeared at the different retention times were fractionated, and structural analysis was performed using LC–MS (Supplementary Fig. S1). The different colored lines reflect different zinc concentrations (0.1, 1 and 5 μM zinc). The number of peaks corresponds to the molecular structure shown in the panel below. (**B**) The production amounts of the three HPLC fractions were quantified, and the amounts of secondary metabolites were compared. The white, black, and gray columns represent gliotoxin, pseurotin A, and fumagillin, respectively. *p < 0.05 indicates a significant difference compared to the control group.

Previously, we reported the role of zinc in gliotoxin biosynthesis and found that ZafA regulates gliotoxin biosynthesis by binding to the *gliZ* promoter in a zinc concentration-dependent manner^45^. To further determine the effect of zinc on the biosynthesis of secondary metabolites, we analyzed culture media of *A. fumigatus* with various zinc concentrations by HPLC. From the HPLC analysis, we found that two other peaks besides gliotoxin appeared in proportion to the zinc concentration. Zinc (0.1, 1, and 5 μM) was added to Czapek-Dox media and cultured for 72 h at 30 °C. The secondary metabolites were purified from the culture media, and HPLC analysis was performed. As shown in Fig. 1A, the gliotoxin peak appeared mainly when the zinc concentration was 0.1 µM and was decreased in proportion to the zinc concentration. With 1 µM zinc, peaks 2 and 3 appeared, and these peaks were fractioned for LC–MS analysis, as shown in Supplementary Fig. S1; these two peaks represented pseurotin A and methylthiogliotoxin, respectively. Interestingly, at 5 μM zinc, the methylthiogliotoxin peak disappeared, and the pseurotin A peak remained in the chromatogram. Previously, the methylthiogliotoxin levels were shown to be increased by zinc^18^. However, the effect of zinc on pseurotin biosynthesis has not yet been reported. To compare the production of secondary metabolites under different zinc concentrations, we quantified the resulting amounts of gliotoxin, pseurotin A, and fumagillin, as shown in Fig. 1B. As described above, the biosynthesis of gliotoxin and pseurotin A tend to oppose each other. These results indicate that the biosynthesis of secondary metabolites is regulated by metals, especially zinc, and the biosynthesis of gliotoxin and pseurotin A are closely related to each other. We further investigated the effect of zinc on the biosynthesis of gliotoxin and pseurotin A and the relationship between gliotoxin and pseurotin A.

### Pseurotin A biosynthesis and the expression of genes involved in pseurotin A biosynthesis were increased by high-zinc conditions

**Figure 2:**
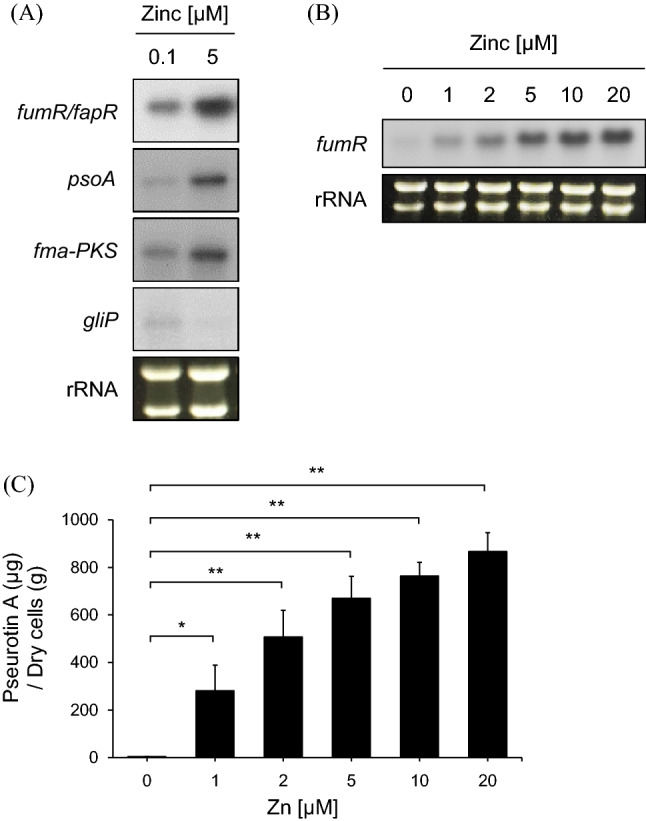
The genes involved in pseurotin A biosynthesis were regulated by zinc. (**A**) The genes shown in the microarray data were confirmed by northern blotting. The cells were cultured in Czapek-Dox media with the indicated zinc concentration, and total RNA was extracted to perform northern blotting. The loading control was the rRNA. (**B**) The zinc dependence of the expression of *fumR* was investigated. The cells were cultured in Czapek-Dox media with the indicated zinc concentration, total RNA was extracted, and northern blotting was performed. Pseurotin A was extracted and measured from the same culture media (**C**). *p < 0.05 and **p < 0.01 indicate a significant difference compared to the control group.

To confirm the effects of zinc on pseurotin A production at the gene expression, we performed a northern blot analysis. As shown in Fig. 2A, northern blotting against the genes involved in pseurotin A biosynthesis was performed, as the same results in the cDNA microarray data, the expression of *psoA*, *fma*-*PKS*, and *fumR* were upregulating under high-zinc condition. However, we found that the expression of *gliP*, which encodes nonribosomal peptide synthetase and catalyzes the first step in the gliotoxin biosynthesis pathway, was downregulated under high-zinc conditions. This result indicated that gliotoxin and pseurotin biosynthesis were regulated in opposite ways. Additionally, we confirmed the effects of zinc on the expression of the genes involved in pseurotin A biosynthesis and the production of pseurotin A. As shown in Fig. 2B, the cells were cultured in Czapek-Dox media with the indicated zinc concentration, and then, the total RNA was extracted. Northern blotting was performed against *fumR*. The expression of *fumR* was increased in proportion to the zinc concentration, and the production of pseurotin A was also increased in proportion to the zinc concentration (Fig. 2C). Interestingly, pseurotin A production increased quickly in response to zinc, although the gene expression of *fumR* increased gradually in response to zinc. This discrepancy should be further examined. These data indicate that pseurotin A biosynthesis depends on zinc by upregulating the expression of *fumR*, which is a transcriptional activator.

### Pseurotin A biosynthesis and the expression of the genes involved in pseurotin A biosynthesis were regulated by ZafA

**Figure 3:**
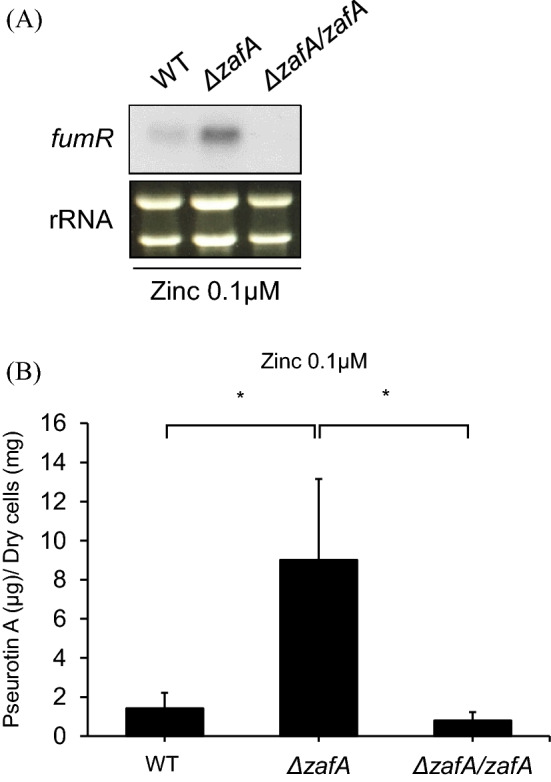
Pseurotin A biosynthesis was regulated by ZafA. (**A**) For determination of the effect of ZafA on *fumR* expression, strains complemented with *zafA* were constructed, and northern blotting was performed against FumR. The indicated strains were cultured in Czapek-Dox media, and total RNA was extracted to perform northern blotting. The rRNA was used as a loading control. (**B**) The biosynthesis pseurotin A was measured using the same culture media. The gray and black columns indicate gliotoxin and pseurotin A, respectively. *p < 0.05 indicates a significant difference compared to the control group.

Next, we also performed northern blot analysis of *fumR* in the *zafA* deletion mutant strain. We used the *zafA* deletion mutant and the *zafA* complementation strain with the *zafA* deletion mutant as reported before (44). A *zafA* complementation strain (*PthiA. zafA*) was constructed by inserting the thiamine-responsive *thiA* promoter (*PthiA*) of *Aspergillus oryzae* into the upstream region of *zafA* (44)*.* Northern blotting was performed against *fumR,* as shown in Fig. 3A. Interestingly, we found that *fumR* expression was upregulated when *zafA* was deleted and that *fumR* expression was downregulated to wild-type levels when *zafA* was introduced into the *zafA* deletion mutant. This result indicates that the expression of *fumR* is regulated by zinc and ZafA. To confirm the northern blot data, we measured pseurotin A production in the *zafA* deletion mutant and the *zafA*-complemented strain of the *zafA* deletion mutant. As shown in Fig. 3B, we found that pseurotin A production increased dramatically when *zafA* was deleted, and this increase in pseurotin A production was decreased to the level of wild-type cells when *zafA* was introduced into the *zafA* deletion strain. And this result is opposite result of gliotoxin production as we reported previously (44). These results indicate that pseurotin A production is regulated by zinc and that the production of pseurotin A and gliotoxin showed opposite regulatory pathways depending on zinc.

### Expression of the genes involved in pseurotin A biosynthesis was regulated by gliotoxin

**Figure 4:**
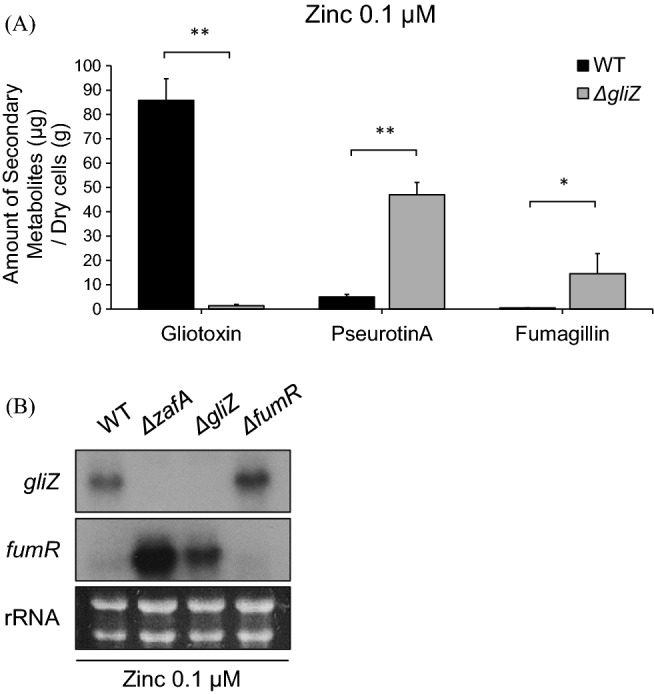
Pseurotin A biosynthesis was regulated by GliZ. (**A**) For identification of the effect of gliotoxin on pseurotin A biosynthesis, pseurotin A was measured in the wild-type and *gliZ* deletion mutant culture media. The indicated strains were cultured in Czapek-Dox media, and secondary metabolites were extracted from the culture media. Then, gliotoxin, pseurotin A, and fumagillin were measured using HPLC. The black and gray columns indicate wild-type and *gliZ* deletion mutants, respectively. *p < 0.05 and **p < 0.01 indicate a significant difference compared to the control group. (**B**) Then, the gene expression levels of *gliZ* and *fumR* were investigated. The wild-type and indicated deletion strains were cultured in Czapek-Dox media, and total RNA was extracted from the cells. Then, northern blotting was performed against *gliZ* and *fumR*. rRNA was used as a loading control.

As described above, the production of pseurotin A and gliotoxin showed opposite effects, and we investigated the relationship between pseurotin A and gliotoxin. We previously reported that ZafA regulates gliotoxin biosynthesis by regulating *gliZ* expression in *A. fumigatus*^44^, and we tested the production of pseurotin A in the *gliZ* deletion mutant. As shown in Fig. 4A, extremely low levels of gliotoxin were detected in the *gliZ* deletion mutant. However, pseurotin A production was increased almost sevenfold compared with that of the wild-type when *gliZ* was deleted. The same result was identified in fumagillin production. To confirm the relationship between pseurotin A production and GliZ, we performed a northern blot analysis with the deletion mutants, as indicated in Fig. 4B. No transcripts of *gliZ* were detected from the *zafA* or *gliZ* deletion mutants, as reported earlier. However, the transcript levels of *fumR* were upregulated when *zafA* and *gliZ* were deleted. Interestingly, the expression level of *fumR* increased to the same level in the *zafA* and *gliZ* deletion mutants, and pseurotin A production also increased in the *zafA* and *gliZ* deletion mutants (Figs. 3B, 4A). These data indicate that pseurotin A biosynthesis is regulated by ZafA and GliZ, although it is not known which of these directly regulates *fumR*. These results indicate that pseurotin A production depends on zinc and is affected by gliotoxin production.

**Figure 5:**
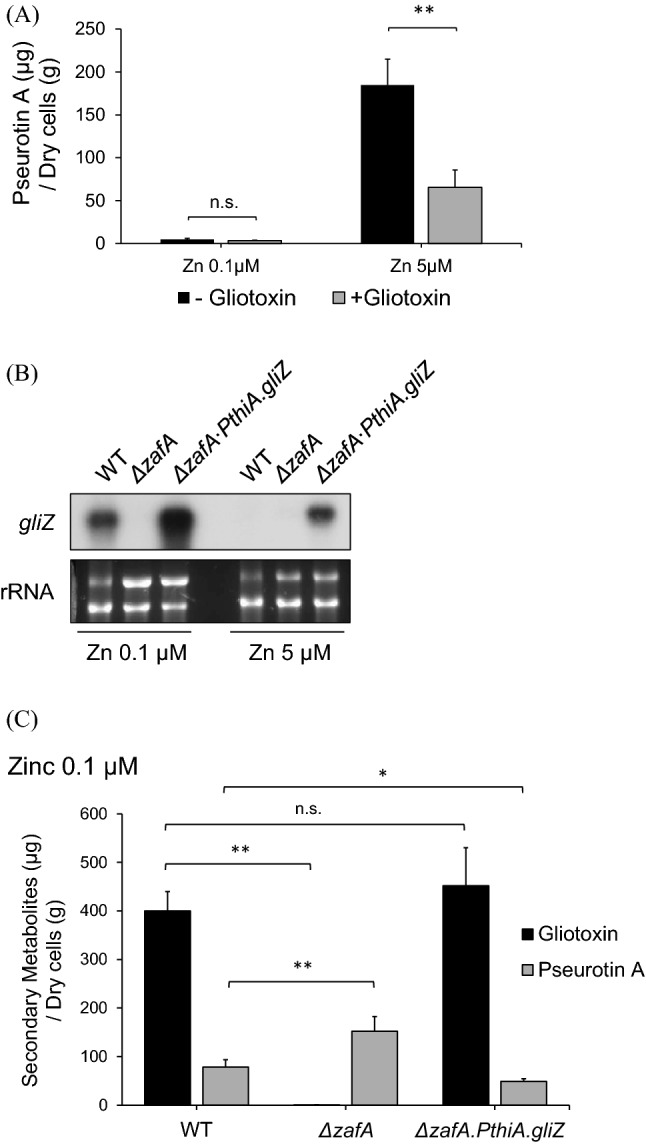
Pseurotin A biosynthesis was regulated by gliotoxin, and ZafA regulated pseurotin A biosynthesis through GliZ. (**A**) For identification of the effect of gliotoxin on pseurotin A biosynthesis, gliotoxin was directly added to the culture media, and pseurotin A was measured. The effect of gliotoxin on pseurotin A biosynthesis was measured under low (0.1 μM) and high zinc (5 μM) conditions. The black and gray columns indicate gliotoxin and pseurotin A, respectively. *ns* Not statistically significant, **p < 0.01 indicates a significant difference compared to the control group. (**B**) For confirmation of the relationship between ZafA and GliZ in pseurotin A biosynthesis, constitutively *gliZ*-expressing cells with the *thiA* promoter in the *zafA* deletion mutant were constructed (Supplementary Fig. S2), and the expression of *gliZ* was confirmed under low (0.1 μM) and high zinc (5 μM) concentrations by northern blotting. The indicated cells were cultured in Czapek-Dox media, and total RNA was extracted from the indicated cells. (C) The amounts of pseurotin A produced in the wild-type, Δ*zafA,* and Δ*zafA∙PthiA.gliZ* strains were measured. *ns *Not statistically significant, *p < 0.05 and **p < 0.01 indicate a significant difference compared to the control group.

To confirm the effects of ZafA and GliZ on pseurotin A production, we investigated the effect of gliotoxin on pseurotin A production, as shown in Fig. 5A. Pseurotin A production decreased by almost 40% in the gliotoxin-treated cells, even though pseurotin A production was very low under low-zinc conditions. The effect of gliotoxin on pseurotin A production was clear under high-zinc conditions. As shown in Fig. 5A, pseurotin A production increased under high zinc concentrations, and treatment with gliotoxin resulted in a decrease in pseurotin A production to 35% of that of the nontreated cells. These data indicate that pseurotin A biosynthesis is negatively regulated by gliotoxin. To confirm the relationship between ZafA and GliZ in pseurotin A biosynthesis, we constructed constitutive *gliZ*-expressing cells with the *thiA* promoter in the *zafA* deletion mutant (Supplementary Fig. S2) and investigated the regulatory mechanisms of ZafA and GliZ in pseurotin A biosynthesis. As shown in Fig. 5B, *gliZ* was highly expressed even under high zinc concentrations in ∆*zafA∙PthiA.gliZ*, resulting from the constitutive expression of *gliZ*. Then, we measured the pseurotin A production amount resulting from the wild-type, ∆*zafA,* and ∆*zafA∙PthiA.gliZ* strains, as shown in Fig. 5C. Gliotoxin production was increased when *gliZ* was overexpressed with *PthiA* even when ZafA was deleted. This result indicates that the ∆*zafA∙PthiA.gliZ* strain highly expresses *gliZ*, and gliotoxin biosynthesis is effective. Interestingly, pseurotin A production was decreased in the ∆*zafA∙PthiA. gliZ* strain, even when *zafA* was deleted, suggesting that ZafA regulates pseurotin A biosynthesis through GliZ.

### Gliotoxin and pseurotin A biosynthesis are virulence factors

**Figure 6:**
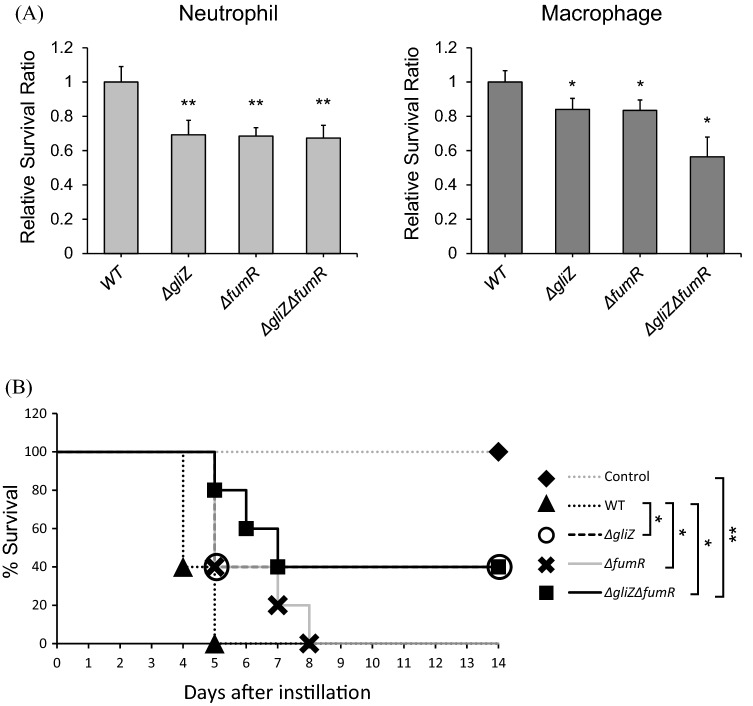
Gliotoxin and pseurotin A biosynthesis are the virulence factors. (**A**) A conidial killing assay with HL-60 differentiated macrophages and neutrophils was performed. A total of 1 × 10^6^ conidia of the indicated strains were added for 1 h, and the infected cells were washed with 1× PBS and harvested with 1× PBS. The cells were resuspended in 100 µl of 1× PBS and lysed, and then, the cell lysates were incubated on AMM. The left panel and right panel indicate neutrophils and macrophages, respectively. The graph shows the relative number of fungal colonies grown on AMM compared to that of the wild-type cells. *p < 0.05 and **p < 0.01 indicate a significant difference compared to the control group. (**B**) A virulence test was performed with a murine model, and 5 × 10^5^ conidia of the indicated strains were used to infect mice. The mice were anesthetized with isoflurane, and 5 × 10^5^ spores (50 µl) were nasally infected per mouse. The number of mice used in each experimental group was five, and all mice were six weeks old. *P* = 0.0078. Curves were plotted and p-values were calculated by using GraphPad Prism software. *p < 0.05 and **p < 0.01 indicate a significant difference compared to the control group.

Gliotoxin and fumagillin are known as virulence factors, and we attempted to determine the additive effects of GliZ and FumR in the pathogenesis of *A. fumigatus*. The conidial killing assay was performed with human macrophage and neutrophil cell lines as shown in Fig. 6A. Differentiated HL-60 neutrophils and macrophage cell lines were infected with the wild-type strain and each mutant strain. *gliZ* and *fumR* single-deletion mutants showed lower viability than wild-type cells, and *gliZ* and *fumR* double-deletion mutants did not show differences from the *gliZ* and *fumR* single-deletion mutants in the differentiated HL-60 neutrophil cell line. However, we found that *gliZ* and *fumR* double-deletion mutants had lower viability than the *gliZ* and *fumR* single-deletion mutants in the differentiated HL-60 macrophage cell line.

To confirm virulence, we performed a murine virulence assay. For the murine infection assay, 6-week-old male BALB/c mice were prepared. Starting on day − 4, the mice were injected with cyclophosphamide (150 mg/kg body weight) once every 3 days to suppress their immune system, and hydrocortisone acetate (112.5 mg/kg body weight) was injected on day − 1. The mice were anesthetized by isoflurane inhalation and treated intranasally with a 20-μl drop of solution containing 5 × 10^5^ conidia. Placing a drop of the spore solution on the nose of anesthetized mice allowed the mice to inhale the solution without pain. Five mice were included in each experimental group and the mouse virulent assay was performed two times independently. As shown in Fig. 6B, the mice infected with wild-type cells started to die 4 days after infection, and all mice died within 5 days. Forty percent of the mice infected with the *gliZ* deletion mutant survived 5 days after infection. However, 80% of the mice infected with the *gliZ* and *fumR* double-deletion mutant survived during the same period, and 60% and 40% of the mice survived for 6 and 7 days after infection, respectively. This result confirmed that the mice infected with the *gliZ* and *fumR* double-deletion mutants exhibited delayed mortality compared with the mice infected with the wild-type and *gliZ* single deletion mutants. Conidia killing and murine virulence assays showed the additive effects of *gliZ* and *fumR* on virulence. These results indicate that GliZ and FumR have important functions in fungal infection.

## Discussion

When microbial pathogens infect mammalian hosts, neutrophils attack microbial pathogens first^4,6^ and phagocytosis occurs. In the phagocytosome, diverse antimicrobial factors, such as extracellular protease, redox enzymes, and bioactive molecules, are produced to inhibit or eliminate microbial pathogens^46,47^. Nutritional immunity occurs through reductions in zinc and copper concentrations; these metals are trace elements but are essential for microbial survival^9,45^. For example, if the host depletes zinc during phagocytosis, microbial pathogens cannot use zinc as a nutrient, and microbial growth is ultimately inhibited^7,13^. However, microbial pathogens overcome zinc depletion by activating the expression of genes involved in zinc uptake. This phenomenon has been reported and studied in many microbial pathogens.

Microbial pathogens also employ diverse strategies to protect themselves against host attacks, and the biosynthesis of secondary metabolites is one of these strategies used by fungal pathogens. *A. fumigatus* produces diverse secondary metabolites, and each secondary metabolite is synthesized by an enzymatic reaction mediated by several fungus-specific genes that are clustered in the genome^48^. Gliotoxin is a well-studied secondary metabolite produced by *A. fumigatus*, and the ability of this fungus to biosynthesize gliotoxin is a virulence factor^49^. Gliotoxin is synthesized by several enzymes encoded by specific genes clustered in the genome, and *gliZ* encodes a transcriptional activator for gliotoxin biosynthetic genes and regulates gene expression involved in gliotoxin biosynthesis. The *gliZ-*deleted strain failed to synthesize gliotoxin and partially lost virulence in the murine virulence test^40^. Interestingly, the biosynthesis of gliotoxin is regulated by zinc utilization and regulated by ZafA, which is a zinc-responsive transcriptional activator^43,44^. This finding indicates that the biosynthesis of zinc and gliotoxin is very closely linked.

In this report, we identified another secondary metabolite, pseurotin A, that is regulated by zinc. Pseurotin A is an antioxidant and inhibits osteoclastogenesis by suppressing reactive oxygen species in vitro and in a mouse model^50^. Interestingly, the production of pseurotin A increased in proportion to the zinc concentration, although it quickly became saturated and showed the opposite pattern to the regulation of gliotoxin. Here, we identified the relationship between gliotoxin and pseurotin A production in *A. fumigatus*. As shown in Figs. 1 and 2, we confirmed the effect of zinc on pseurotin A biosynthesis, which is regulated at the transcriptional level. Furthermore, gliotoxin affects pseurotin A production at the transcriptional level via GliZ. As we previously described the relationship between gliotoxin and zinc, zinc clearly regulates gliotoxin biosynthesis at the transcriptional level. Gene expression of *gliZ* was not affected by FumR (Fig. 4B). Furthermore, pseurotin A biosynthesis was not affected by ZafA in cells constitutively expressing GliZ. These results indicate that zinc regulates pseurotin A biosynthesis through GliZ, but the reverse pathway is not viable.

Four questions are raised by these results. The first question concerns why gliotoxin and pseurotin A are regulated by opposite pathways. Zinc utilization in neutrophils is a well-studied type of nutritional immunity, and gliotoxin is affected by zinc starvation. Under zinc starvation conditions in neutrophils, *A. fumigatus* upregulates the expression of zinc transporters by ZafA, and gliotoxin is increased. We believe that gliotoxin is sufficient to combat host protection and that it is not necessary to synthesize pseurotin A. Thus, gliotoxin inhibits pseurotin A biosynthesis. The second question involves why pseurotin A is regulated by gliotoxin metabolism. As we described, FumR regulates both the pseurotin A and fumagillin biosynthesis pathways, and fumagillin biosynthesis has been reported to be involved in the pathogenesis of *A. fumigatus*^51^. Although our report is focused on pseurotin A biosynthesis, fumagillin biosynthesis is also regulated in a gliotoxin-negative manner. This result indicates that FumR is also involved in pathogenesis and that *gliZ* and *fumR* double deletion resulted in additive effects on conidial infections and murine death rates (Fig. 6). Here, we suggest that gliotoxin and pseurotin A are important in pathogenesis and show redundancy; in other words, one of them is required in pathogenesis, and gliotoxin may function first, sequentially followed by pseurotin A or fumagillin. Therefore, gliotoxin inhibits the biosynthesis of pseurotin A or fumagillin, and then, pseurotin A and fumagillin are synthesized when gliotoxin fails. The third question involves the functions of pseurotin A during fungal infection. During fungal infection, macrophages produce high levels of ROS, and pseurotin A may suppress them. This hypothesis coincides with the fact that pseurotin A is an antioxidant and that the production of pseurotin A was increased under high-zinc conditions, which can induce ROS formation.

**Figure 7:**
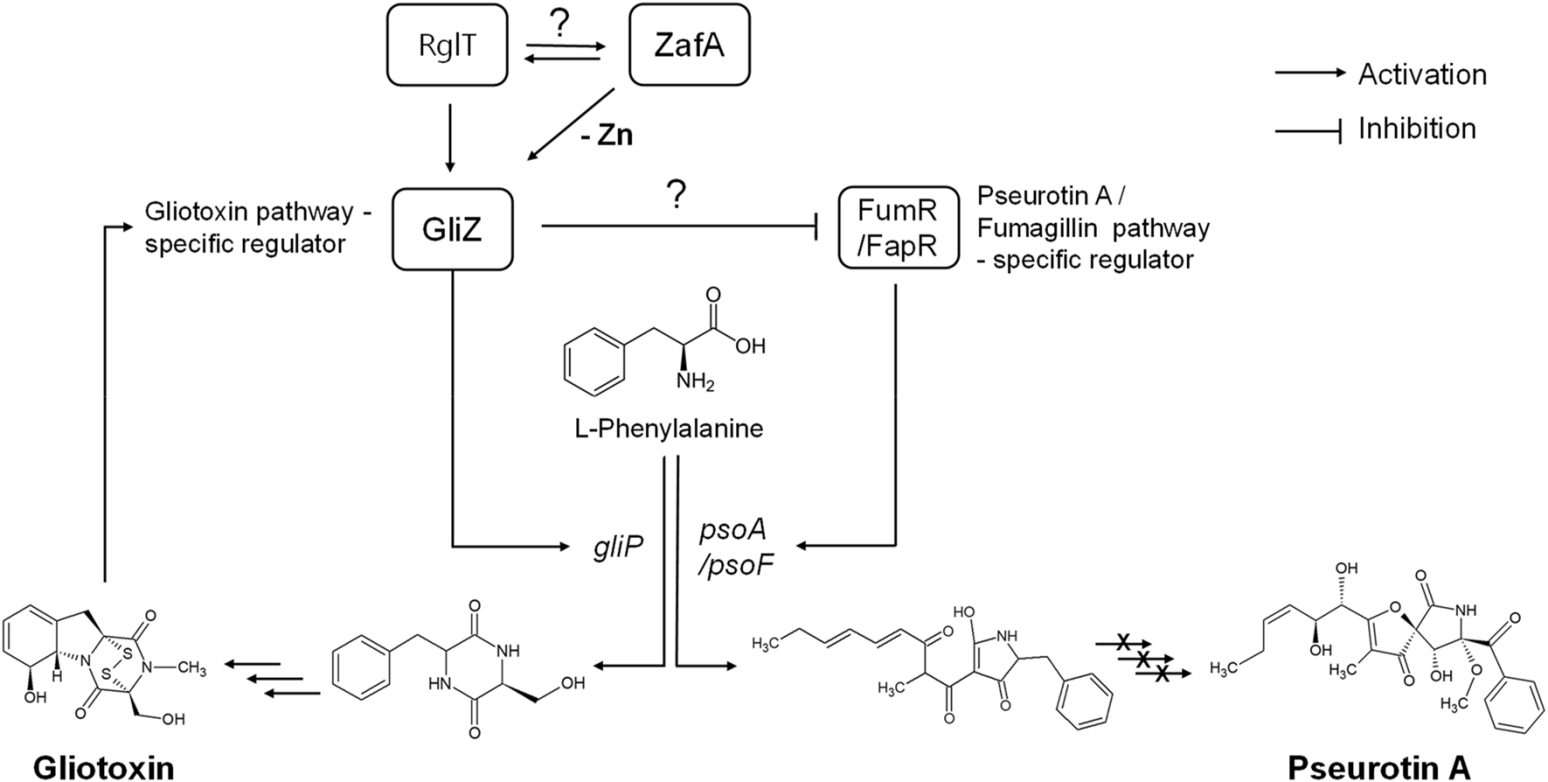
The proposed regulatory model of pseurotin A biosynthesis by ZafA and GliZ.

The fourth and most important question is how GliZ regulates pseurotin A biosynthesis. Pseurotin A biosynthesis is regulated by FumR at the transcriptional level to express pseurotin A biosynthetic genes, and identifying the regulatory mechanism of FumR by GliZ would answer this question. To identify this regulatory mechanism, we performed EMSA to determine whether GliZ binds to the 5' upstream region of *fumR*. As we described, we identified the regulatory mechanisms by which zinc, GliZ, and FumR negatively regulate pseurotin A biosynthesis sequentially (Fig. 7), and we tried to perform EMSA with purified GliZ. However, we did not find binding motifs with GliZ from the 5' upstream region of *fumR* by EMSA (data not shown). Additionally, we tried to find a ZafA binding motif from the 5' upstream region of *fumR*, and we did not find this motif (data not shown). These results indicate that GliZ does not directly regulate pseurotin A biosynthesis; rather, other secondary mechanisms are involved in regulation by GliZ because our results clearly showed the GliZ dependency of pseurotin A biosynthesis. Recently, it has been reported that RglT has an important function in gliotoxin biosynthesis and self-protection^52^. RglT regulates the expression of Gli gene cluster and the biosynthesis of gliotoxin. Interestingly, Δ*rglT* strain showed growth defect when zinc chelator phenanthroline was treated^53^. This report indicates that RglT is involved in zinc metabolism and gliotoxin biosynthesis. The detailed regulatory mechanism should be uncovered in the future.

Secondary metabolites are common in the microbial world, and fungi also produce diverse secondary metabolites^16^. Although many secondary metabolites have important roles in the treatment of diseases^54^, some of them are toxic to living organisms and serve as virulence factors of pathogenic fungi^14^. *A. fumigatus* produces 19 putative secondary metabolites, and the genes involved in the biosynthesis of each of these secondary metabolites are clustered at specific loci of fungal chromosomes^55^. Interestingly, we showed that the biosynthesis of gliotoxin is regulated by zinc utilization and that gliotoxin also regulates pseurotin A biosynthesis, even though the detailed regulatory mechanism has not yet been identified. Our data indicate that the biosynthesis of secondary metabolites is closely linked, and these results will aid in controlling secondary metabolite biosynthesis, especially in cases of toxins.

## Methods

### Strains, media and growth conditions

The fungal strain used in this study was the *A. fumigatus* A1163 strain. A *gliZ* (AFUB_075680) deletion strain (*∆gliZ*) was constructed by transforming the deletion cassette vector containing the 5'-UTR and 3′-UTR of the gene and the *pyrG* gene as a selection marker to auxotrophs of *A. fumigatus*. The wild-type and its derivatives were maintained on Aspergillus minimal medium (AMM, glucose, salt mix, magnesium sulfate, Hunter’s trace element solution, agar) or complete medium (CM) agar plates. The medium used to induce secondary metabolites was Czapek-Dox medium^56^. Zinc sulfate (0.1, 1 and 5 μM) or exogenous gliotoxin (2 μg/ml) was added to the media when necessary.

To generate *ΔgliZ, ΔfumR,* and *ΔzafA.PhiA-gliZ* strains, we constructed various cassettes based on the pRS1190 vector containing *pyrG* as a selection marker and constructed them as described previously^57,58^. The constitutively expressed *gliZ* (*ΔzafA*. *PhiA*- *gliZ*) strain was constructed based on *ΔzafA,* and *pyrG* was the pop-out. To generate the *gliZ* cassette (*PthiA*-*gliZ* cassette), we introduced the end of AFUB_075670 into one side of the *pyrG* marker, and the front part of the *gliZ* gene was linked to the thiamine-responsive *thiA* promoter (*PthiA*) of *A. oryzae* on the other side. This *gliZ* cassette was transformed to the *ΔzafA* and *pyrG* pop-out strains. The strain was confirmed by southern blotting. The primer sets used in this study are summarized in Supplementary Table S1.

### Secondary metabolite extraction and HPLC analysis

Secondary metabolites were extracted from the culture supernatant of *A. fumigatus*, which was cultured in non-Czapek-Dox media for 72 h. The extraction procedures of secondary metabolites were performed as described previously^56^. These samples were analyzed using reverse-phase high-performance liquid chromatography (RP-HPLC) with a UV detector and a C18 column (Agilent Eclipse XDB-C18 (5 µm) 4.6 × 250 mm) at a 1 mL/min flow rate. The mobile phase-gradient system for HPLC was carried out as described in Supplementary Table S2. For detection of gliotoxin, pseurotin A and fumagillin, wavelengths of 254 nm and 351 nm were used.

### RNA extraction and Northern blot

Northern blotting was performed as described by Sambrook and Russell^59^. Total RNA was obtained from *A. fumigatus* by using TRIzol® (Invitrogen, Seoul, Korea). The amount of RNA loaded in each lane was 5 or 10 µg. The separated RNAs on the gel were transferred to a nylon membrane (Hybond-N + nylon membrane, GE Healthcare), and northern blotting was performed. The PCR products amplified from the exon regions of target genes were used as northern blot probes and labeled with a-[^32^P] radioactive dCTP using a random primer kit (GE Healthcare, Seoul, Korea). The ribosomal RNA was used as the loading control.

### LC–MS analysis

LC–MS analysis of the purified HPLC fractions of secondary metabolites was performed using liquid chromatography coupled to triple quadrupole mass spectrometry (TSQ Quantum Ultra EMR, Thermo Fisher Scientific, San Jose, CA) at the Korea Basic Science Institute (Seoul, Korea) as described previously^60^. The samples were examined using flow-injection analysis. The MS spectrum was obtained in negative (positive) full scan mode (*m/z* 100–600). The mass parameters were as follows: spray voltage of 3 kV (4 kV), sheath gas pressure of 40 (arbitrary unit), aux gas pressure of 10 (arbitrary unit) and capillary temperature of 300 °C. For data acquisition and processing, Xcalibur 2.1.0 software (Thermo Fisher Scientific, San Jose, CA, USA) was used.

### Conidial killing assay

HL-60 cells were cultured in RPMI 1640 medium containing 10% fetal bovine serum (FBS) and 1% Gibco™ antibiotic–antimycotic at 37 °C with 5% CO_2._ To induce the differentiation of HL-60 cells into macrophage-like and neutrophil-like cells, we treated the cells with 50 nM 12-o-tetradecanoylphorbol-13-acetate (PMA) and 1.25% dimethyl sulfoxide (DMSO) respectively for 5 days. The macrophage induction is confirmed by observing the cell shape changed to a radial shape and attached to the bottom of the cell dish. Neutrophil differentiation was carried out by confirming under a microscope whether the number of cells containing multilobed nuclei was about 70–80% of the total cells through DAPI staining. A total of 10^3^ swollen conidia were infected with 5 × 10^4^ differentiated HL-60 cells in RPMI 1640 medium supplemented with 5% FBS. After incubation for 0, 1, and 2 h, the samples were vortexed vigorously, and the numbers of surviving fungal cells were determined. Swollen conidia in the absence of differentiated HL-60 cells were processed as a negative control. The results were processed using Student’s t-test for unpaired samples, and P-values less than 0.05 were considered significant.

### Mouse virulence assay

The wild-type and mutant *A. fumigatus* strains were incubated for 5 days at 37 °C on agar AMM, and the spores were harvested using 0.9% NaCl solution containing 0.01% Tween 80. The concentration of spores was adjusted to 2.5 × 10^7^ spores/mL. For the murine infection assay, six-week-old male mice (BALB/c strain; body weight 18–20 g) were purchased from Doo-Yeol Biotech Co. (Seoul, Korea). Starting on day − 4, the mice were injected with cyclophosphamide (150 mg/kg body weight) once every 3 days to suppress their immune system, and hydrocortisone acetate (112.5 mg/kg body weight) was injected on day − 1. The mice were anesthetized by inhaling isoflurane and treated intranasally with a 20 μl drop of solution containing 5 × 10^5^ conidia. Putting a drop of the spore solution on the nose of anesthetized mice allowed the mice to inhale the solution without pain. The number of mice used in each experimental group was five. To prevent bacterial infection, 1 g/l of tetracycline was added to the drinking water. The mice were monitored daily. The survival rate was calculated by counting the number of mice that survived each day. At 14 days, all mice were humanely euthanized using CO_2_ gas chamber according to the IACUC guidelines, Korea University. All animal experiments were performed in the Central Laboratory Animal Research Center, Korea University, Seoul, Korea. Experiments were repeated in triplicate.

### Ethics statement

All animal experiments were conducted in accordance with the Guidelines for the Care and Use of Laboratory Animals outlined by the Ministry of Food and Drug Safety, Republic of Korea. All animals were grown in ventilated individual cages in accordance with the protocols approved by the Institutional Animal Care and Use Committee of Korea University (permit No. KUIACUC-2011-139) and were humanely euthanized using CO_2_ gas chamber according to the IACUC guidelines, Korea University. Additionally, this study was carried out following the recommendations of the ARRIVE Guidelines.

### Statistical analysis

All experiments, including the conidial killing assay and production of secondary metabolites, were performed in triplicate. The mouse virulence assay was performed as two independent experiments. The differences among the groups were assessed using Student’s t-test for unpaired samples, and P-values less than 0.05 were considered significant. Statistical significance was marked with asterisks. One asterisk (*) indicates that the P-value is less than 0.05, and two asterisks (**) indicate a P-value less than 0.01.

## Supplementary Information

Supplementary Information 1.

Supplementary Information 2.

Supplementary Information 3.

Supplementary Information 4.

Supplementary Information 5.

Supplementary Information 6.

Supplementary Information 7.

Supplementary Information 8.

Supplementary Information 9.

Supplementary Information 10.

Supplementary Information 11.

Supplementary Information 12.

Supplementary Information 13.

Supplementary Information 14.

## Data Availability

All data generated from this work are included in the manuscript and its supplementary files.
