# Supplementary figures and images for: Regulation of pseurotin A biosynthesis by GliZ and zinc in Aspergillus fumigatus
Source: Sci Rep. 2023 Feb 10;13:2431. doi: 10.1038/s41598-023-29753-z (PMC9918513; doi:10.1038/s41598-023-29753-z)

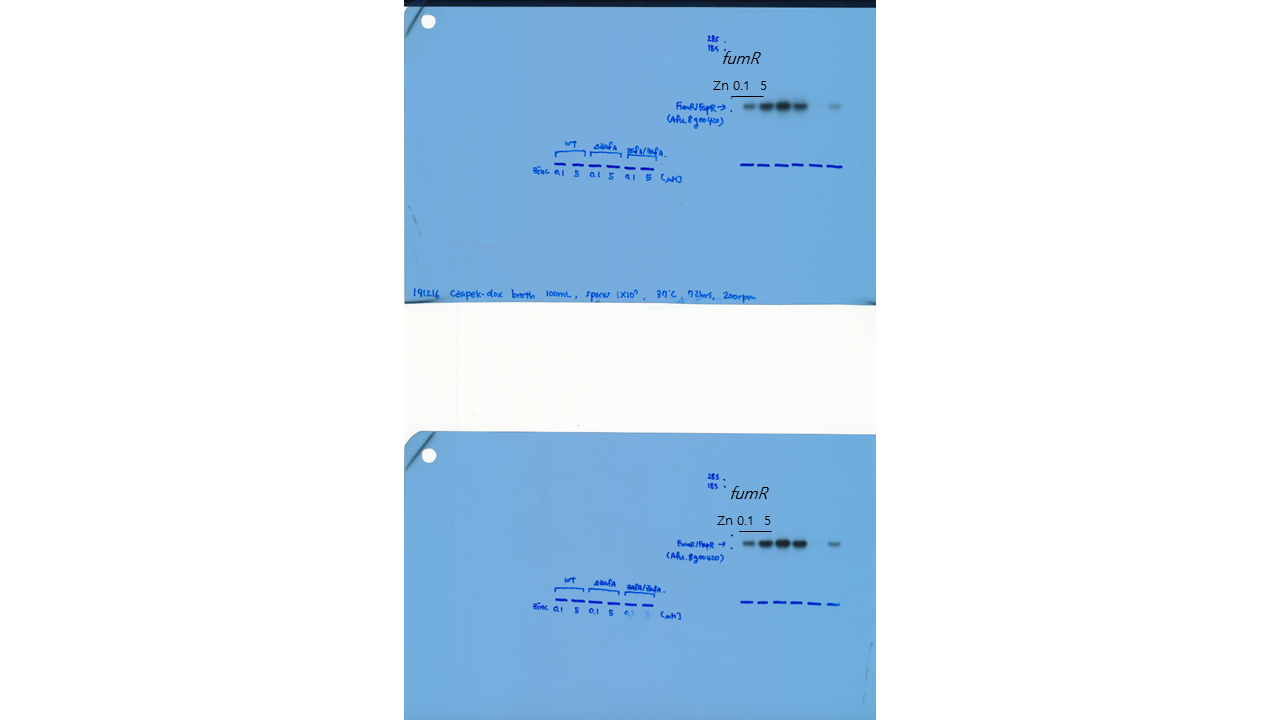

Supplement: Supplementary file 1 — Supplementary Information 1. [file 41598_2023_29753_MOESM1_ESM.tif]

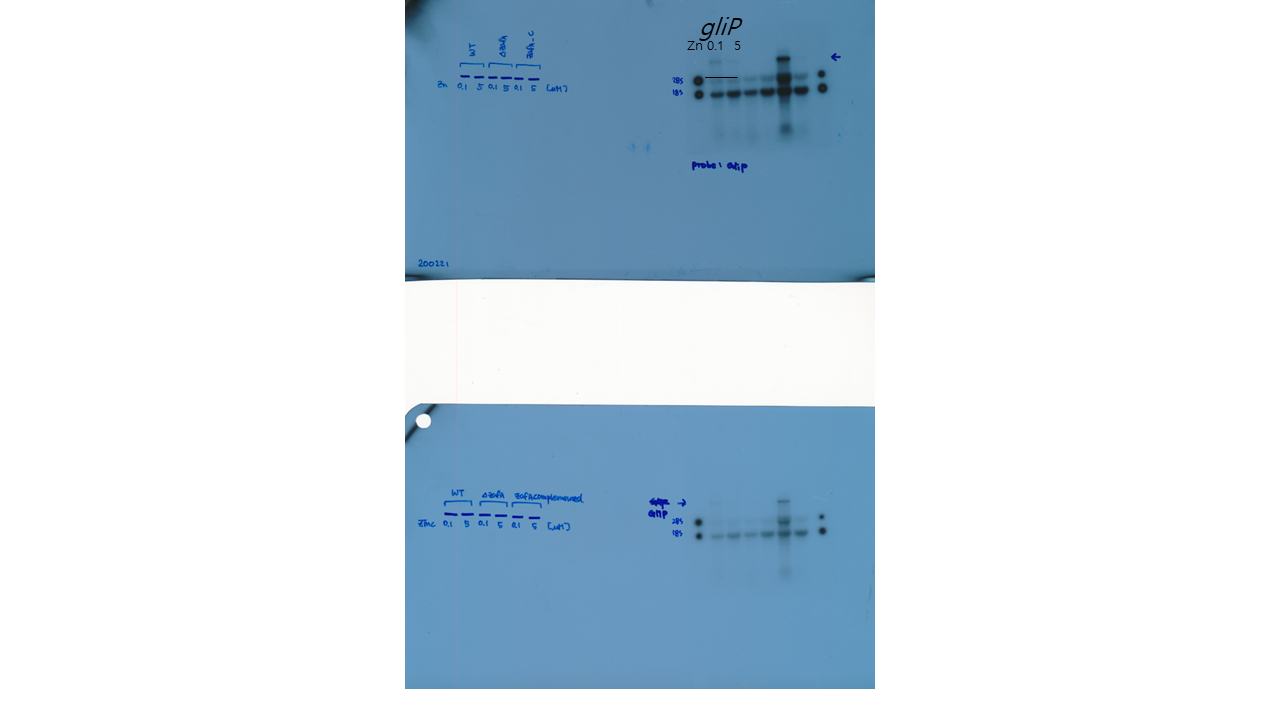

Supplement: Supplementary file 2 — Supplementary Information 2. [file 41598_2023_29753_MOESM2_ESM.tif]

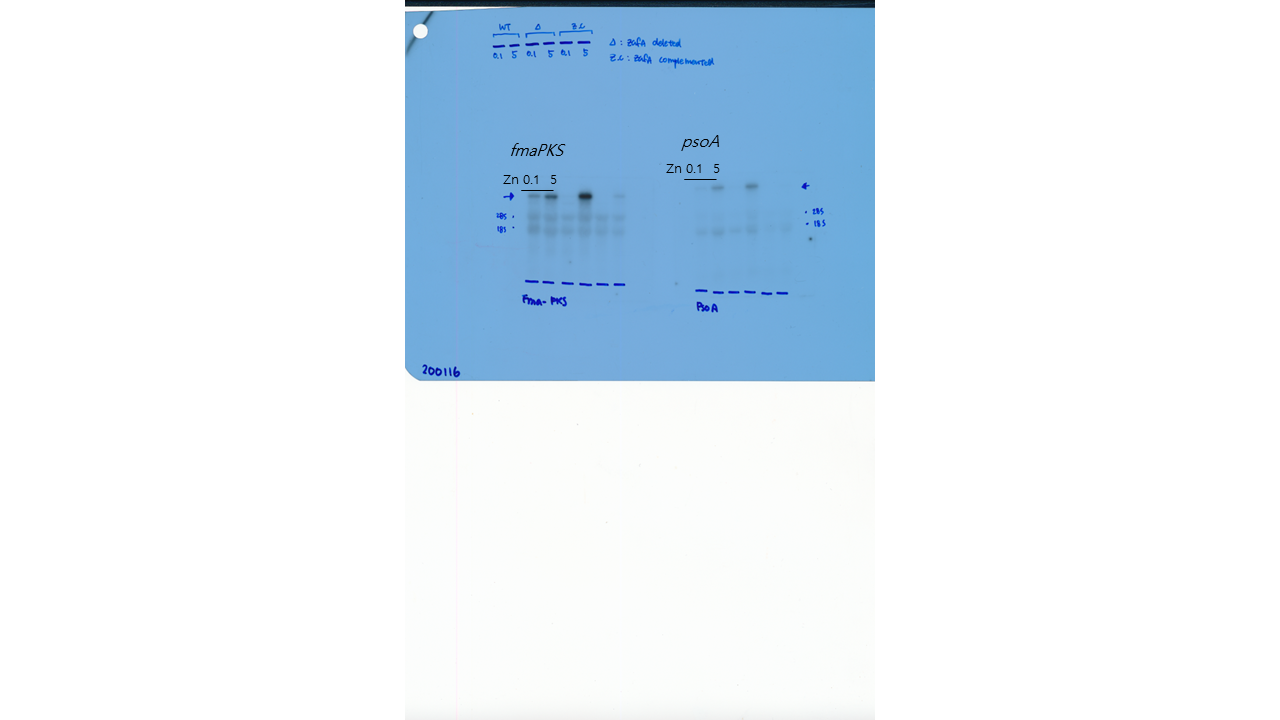

Supplement: Supplementary file 3 — Supplementary Information 3. [file 41598_2023_29753_MOESM3_ESM.tif]

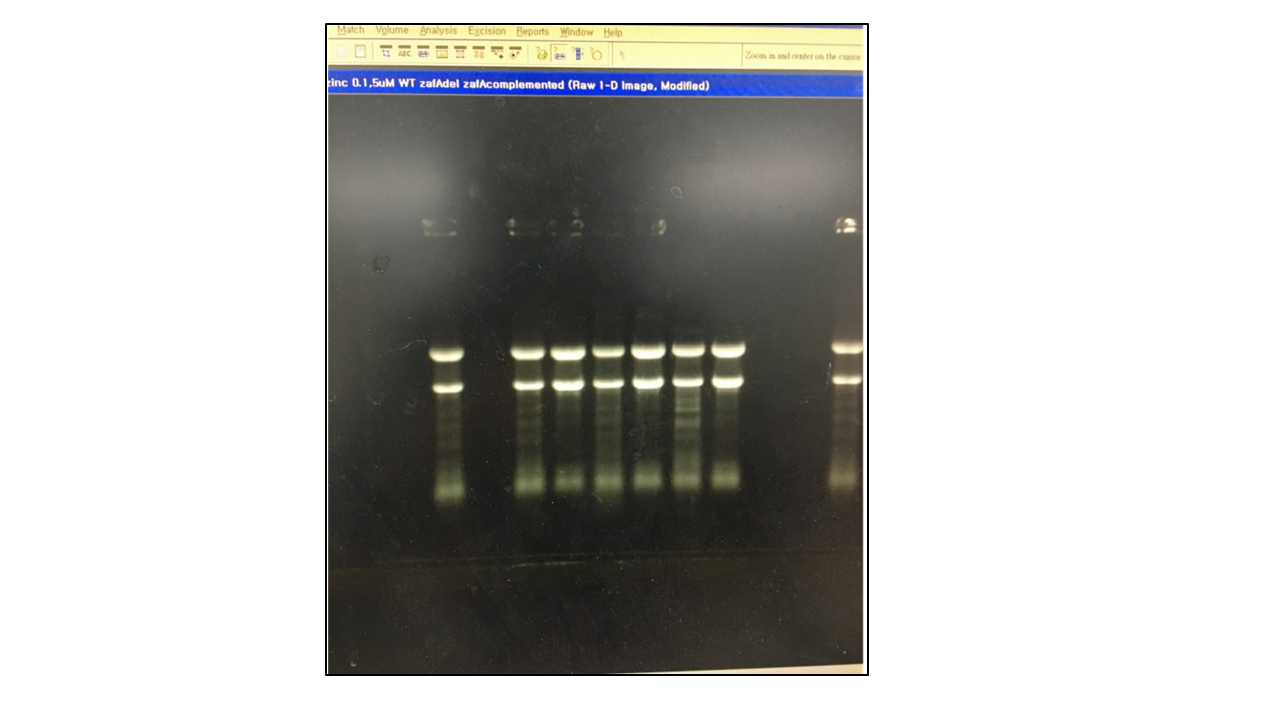

Supplement: Supplementary file 4 — Supplementary Information 4. [file 41598_2023_29753_MOESM4_ESM.tif]

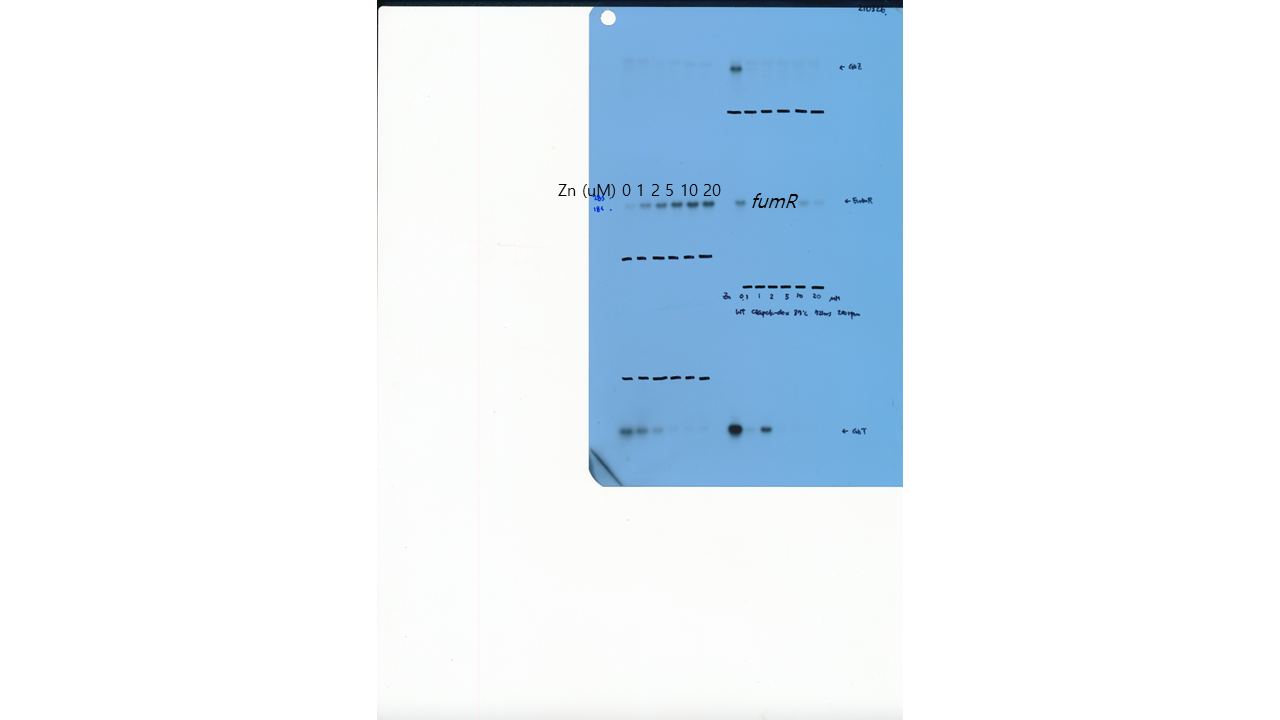

Supplement: Supplementary file 5 — Supplementary Information 5. [file 41598_2023_29753_MOESM5_ESM.tif]

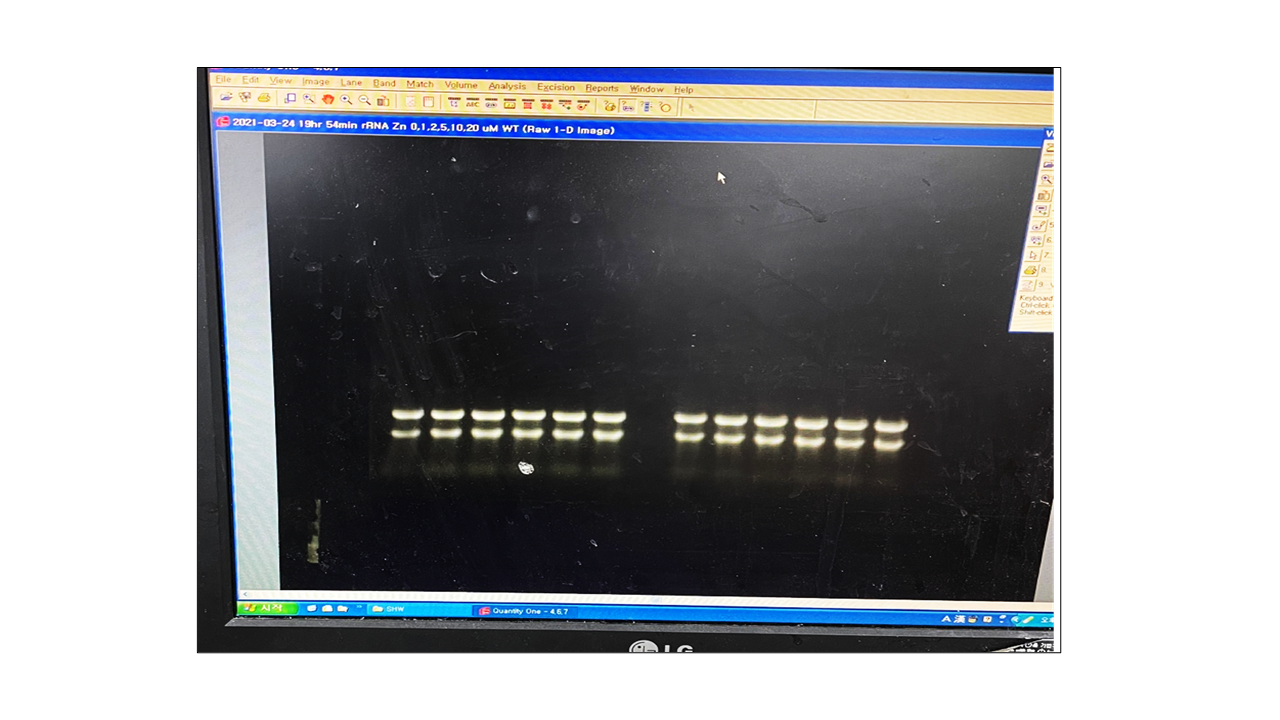

Supplement: Supplementary file 6 — Supplementary Information 6. [file 41598_2023_29753_MOESM6_ESM.tif]

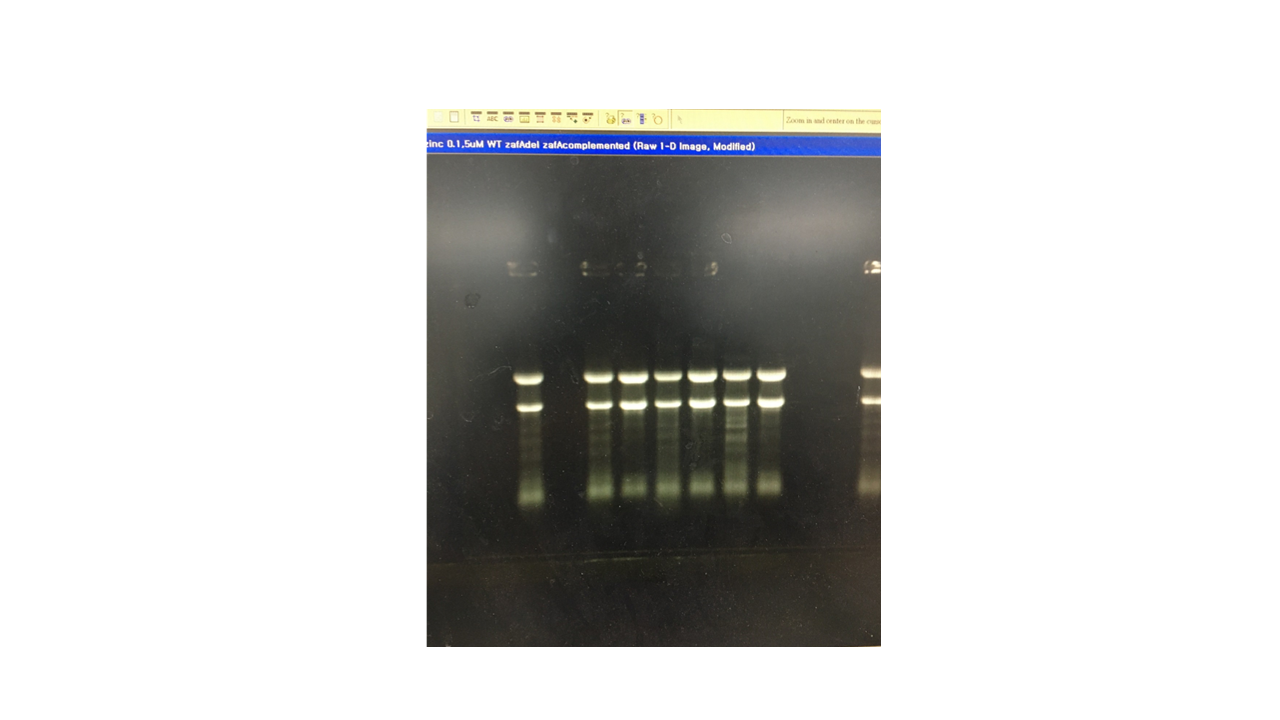

Supplement: Supplementary file 7 — Supplementary Information 7. [file 41598_2023_29753_MOESM7_ESM.tif]

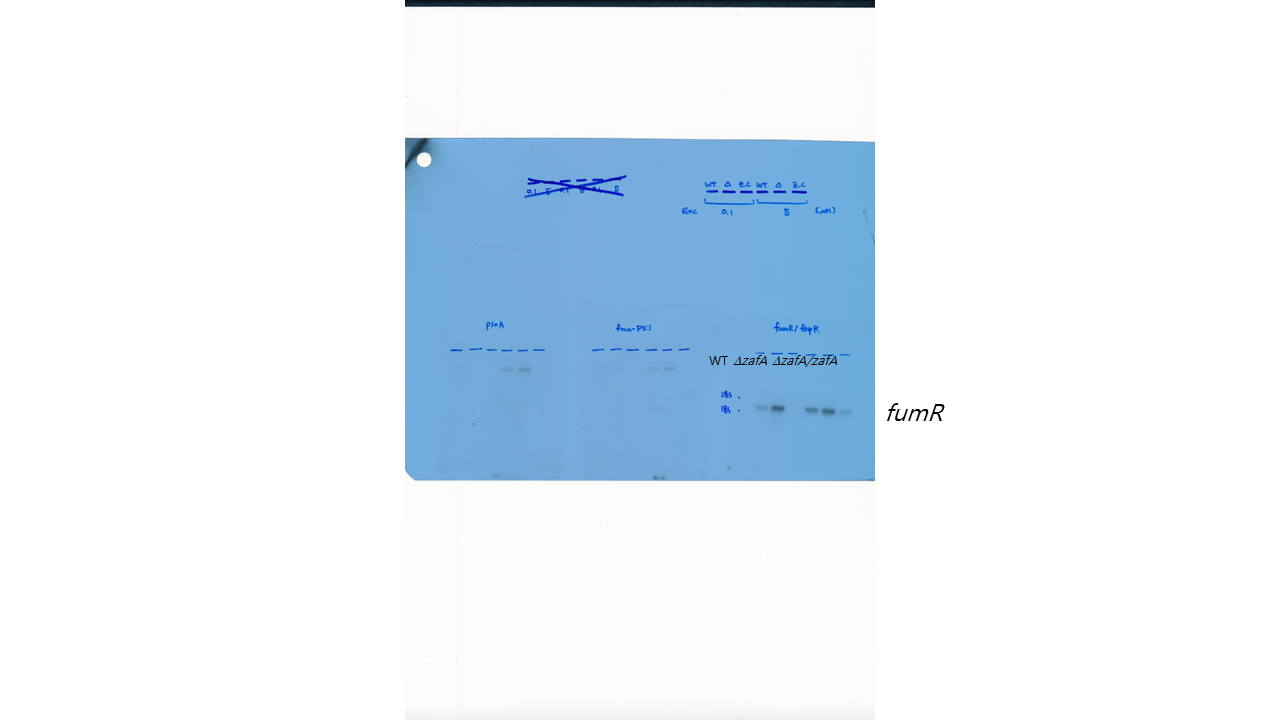

Supplement: Supplementary file 8 — Supplementary Information 8. [file 41598_2023_29753_MOESM8_ESM.tif]

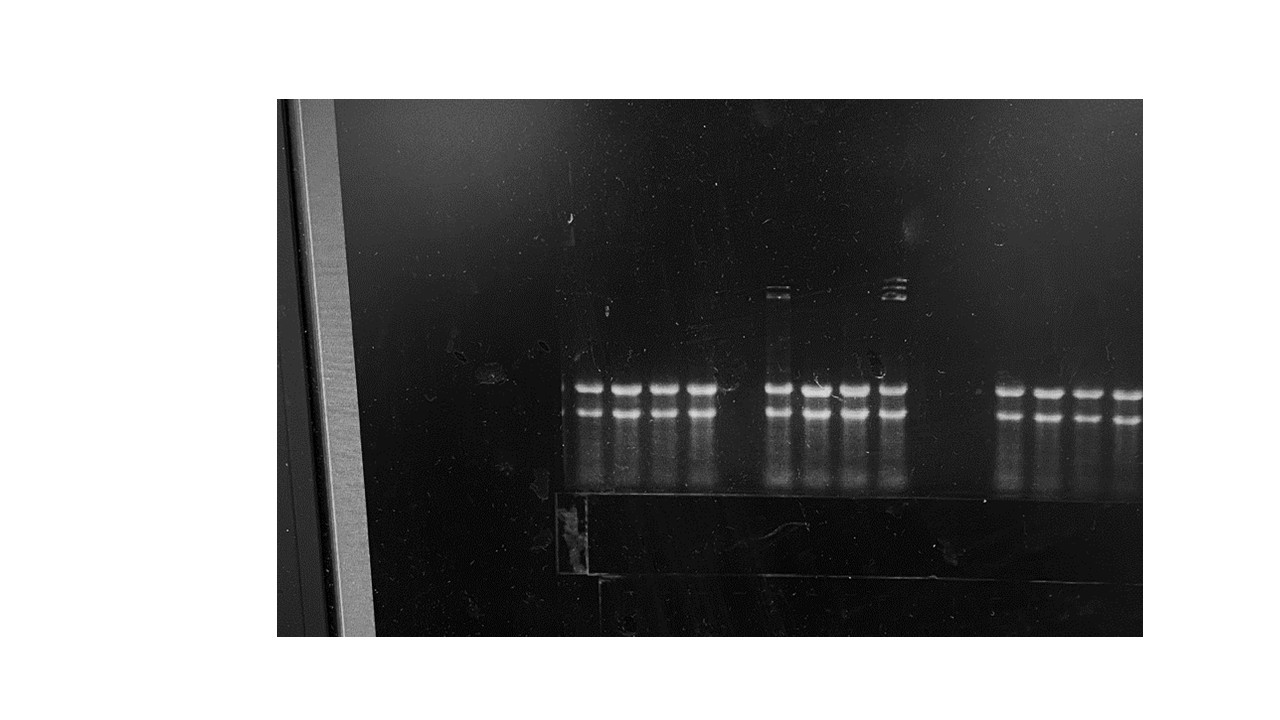

Supplement: Supplementary file 9 — Supplementary Information 9. [file 41598_2023_29753_MOESM9_ESM.tif]

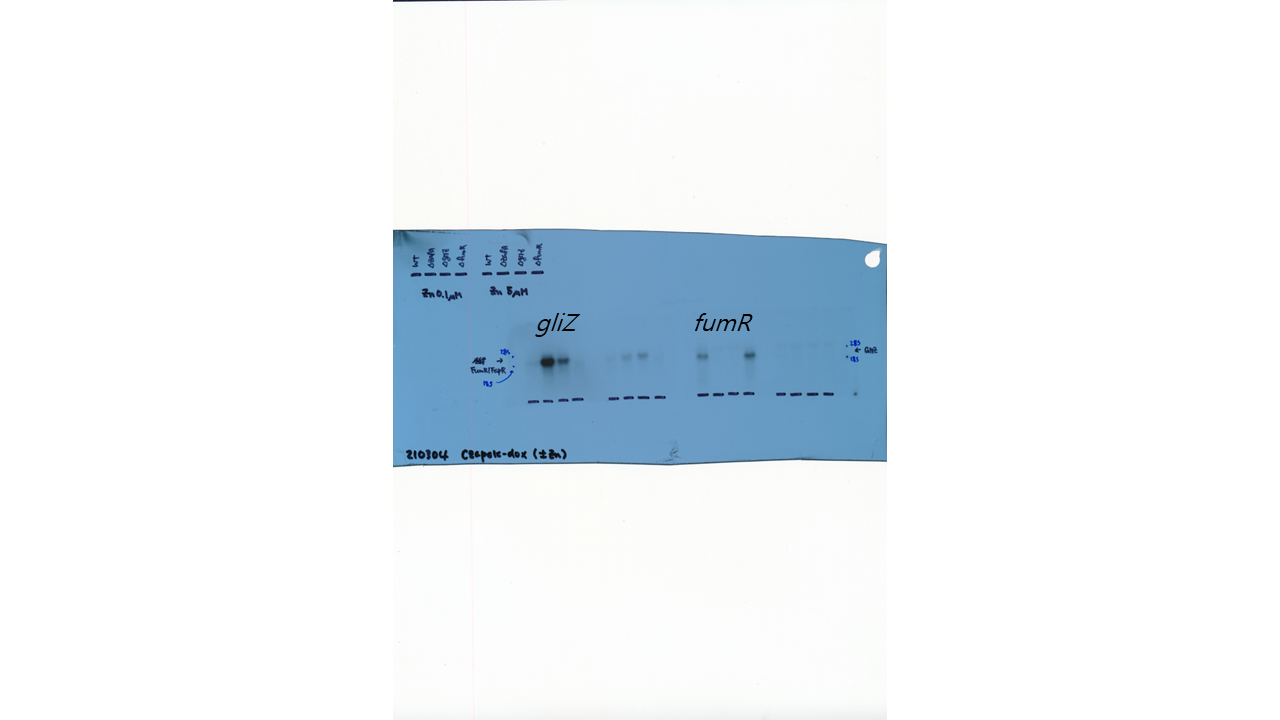

Supplement: Supplementary file 10 — Supplementary Information 10. [file 41598_2023_29753_MOESM10_ESM.tif]

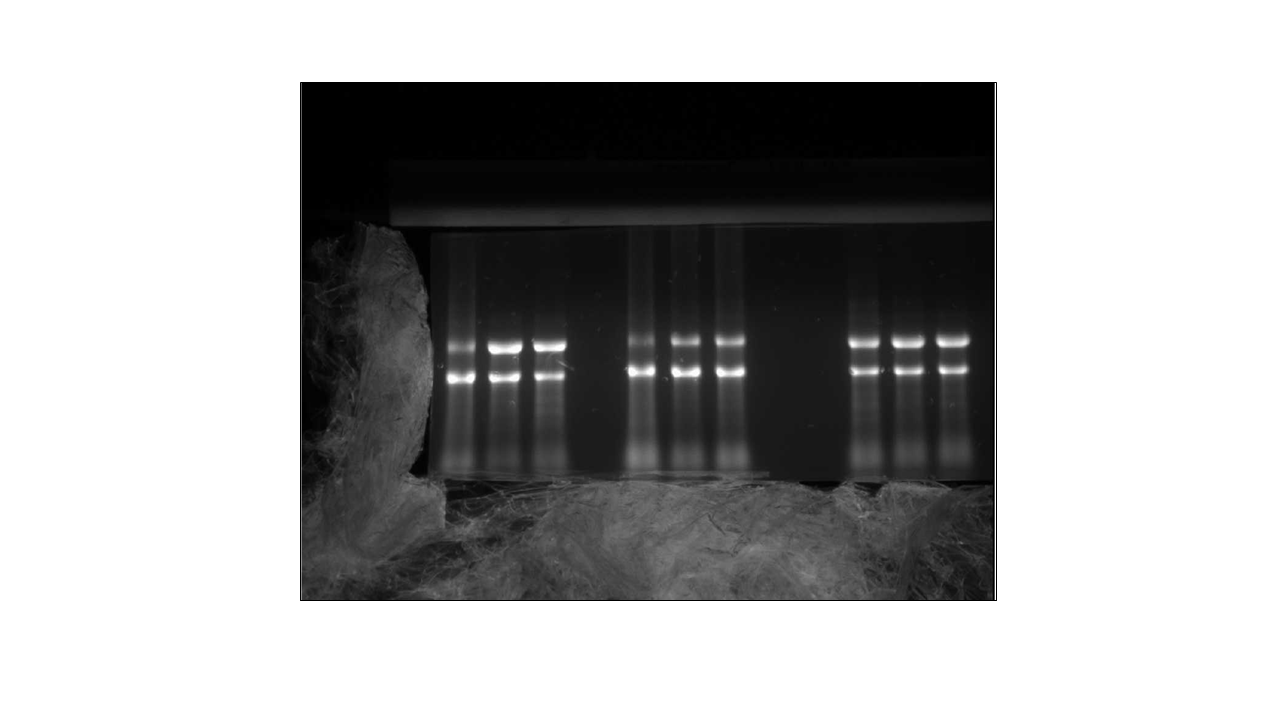

Supplement: Supplementary file 11 — Supplementary Information 11. [file 41598_2023_29753_MOESM11_ESM.tif]

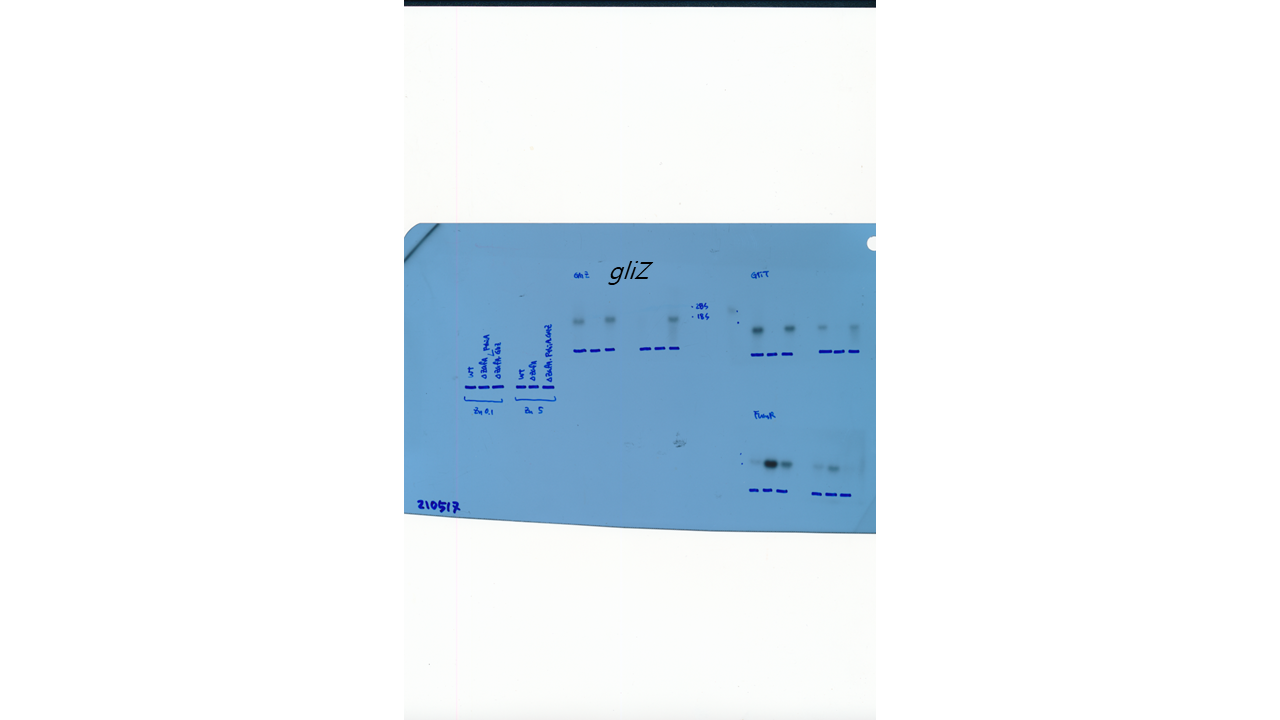

Supplement: Supplementary file 12 — Supplementary Information 12. [file 41598_2023_29753_MOESM12_ESM.tif]

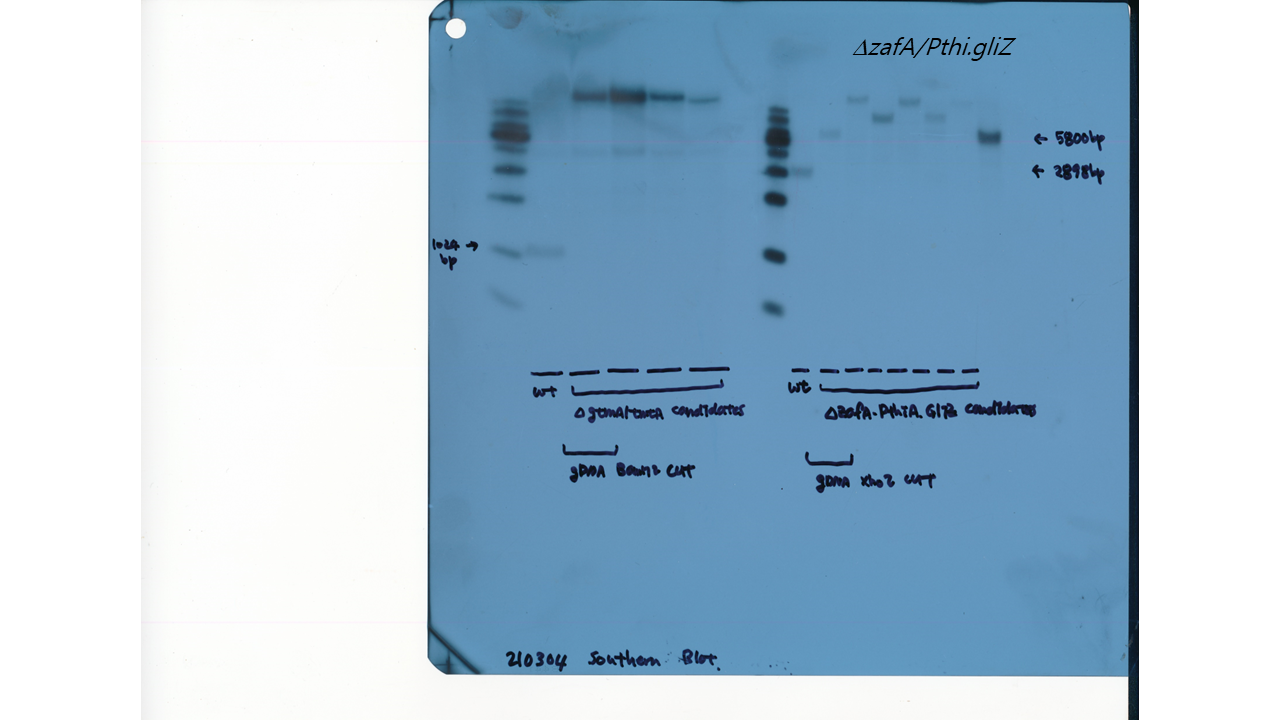

Supplement: Supplementary file 13 — Supplementary Information 13. [file 41598_2023_29753_MOESM13_ESM.tif]
