# Supplementary material for: Regulation of pseurotin A biosynthesis by GliZ and zinc in Aspergillus fumigatus
Source: Sci Rep. 2023 Feb 10;13:2431. doi: 10.1038/s41598-023-29753-z (PMC9918513; doi:10.1038/s41598-023-29753-z)
Supplement: Supplementary file 14 — Supplementary Information 14. [file 41598_2023_29753_MOESM14_ESM.docx]

**Fig. S1. Structural analysis of the HPLC fractions.** The HPLC fractions and standard pseurotin A (Sigma-Aldrich, USA) were analyzed by LC-MS to identify the molecular structure. (A) is the standard pseurotin A and (B) is the fraction of the cell culture extract. The upper panels are HPLC chromatograms, and the lower panels are MS chromatograms of the indicated samples.

**Fig. S2. Construction of constitutive *GliZ*-expressing cells with *ThiA* promoter.**

**Table S1. The primer set used in this study**

**Table S2. Gradient protocols used in the HPLC analysis.**

Fig. S1.

(A)


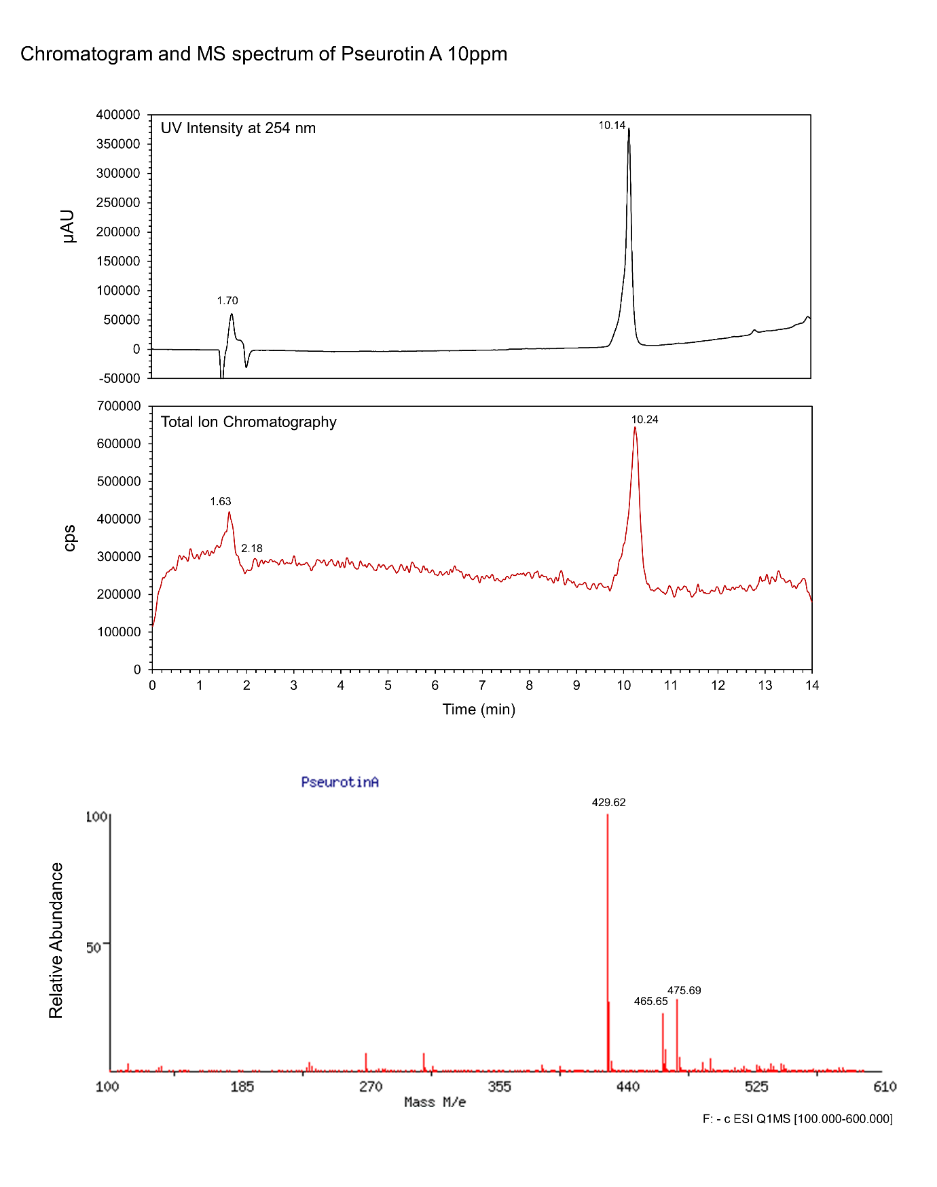


(B)


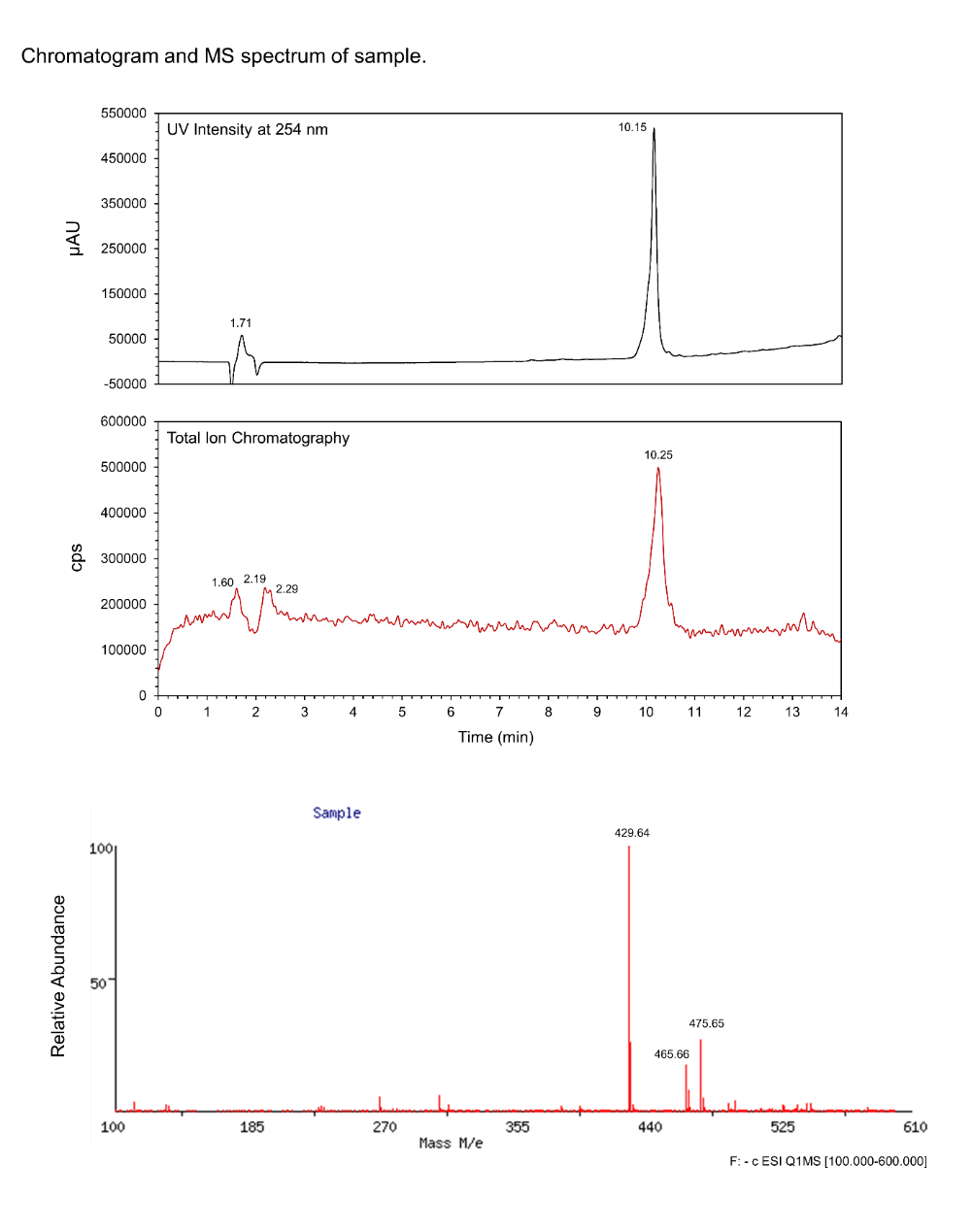


Fig. S2

**Table S1. The primer set used in this study**

|  | Primer name | Sequence (5' → 3') |
| --- | --- | --- |
| Northern blot | AFUB_075680 (GliZ)_north_F | TGCTGCTGCTGCACCCAAGC |
|  | AFUB_075680 (GliZ)_north_R | CGATGTAGCCGGGAGTGAGG |
|  | AFUB_075710 (GliP)_north_F | CTTCGATTCACCTCCCTGTG |
|  | AFUB_075710 (GliP)_north_R | TGTAGCTGAAGGGGAGTCAA |
|  | AFUB_086030 (PsoA)_north_F | TGCCGTTGGATTTGGCTACG |
|  | AFUB_086030 (PsoA)_north_R | CAGCGCTCGATAGCAGCCAG |
|  | AFUB_086200 (Fma-PKS)_north_F | TCCAGAGACTGGTAGCCGTCG |
|  | AFUB_086200 (Fma-PKS)_north_R | CCGATTCCTCTTGGGACGAG |
|  | AFUB_086150 (FumR/FapR)_north_F | GCTTGCTGGATATGCCGACC |
|  | AFUB_086150 (FumR/FapR)_north_R | AAGGAGGAGCGCATCGTAGC |
| *PthiA.GliZ* strain construction & southern blot | PthiA (A.oryzae)_F | GACAGACGGGCAATTGATTACG |
|  | PthiA (A.oryzae)+HindⅢ_R | AAGCTTGTTTCAAGTTGCAATGACTA |
|  | AFUB_075680 (GliZ)_cDNA_hindⅢ_F | AAGCTTATGGCGACAGCTATGCAGGA |
|  | AFUB_075680 (GliZ)_xhoⅠ_R | GGATATCTCGAGCGGTTAGG |
|  | AFUB_075670_F | CCCATAAGGGCGGTGTCATC |
|  | AFUB_075670_R | CTATCGAAAATCTGCCGGTC |
|  | AFUB_075680 (GliZ)_south_F | TGCTGCTGCTGCACCCAAGC |
|  | AFUB_075680 (GliZ)_south_R | CGATGTAGCCGGGAGTGAGG |
| Deletion strain construction | AFUB_075680 (GliZ)_+xhoⅠ_5del_F | CTCGAGGCGGTTGACTGATATCCCTA |
|  | AFUB_075680 (GliZ)_+HindⅢ_5del_R | AAGCTTCGCTGACGAGTAGTTTGCTC |
|  | AFUB_075680 (GliZ)_3del_F | GCTGTTCTCACCTCTTTTTT |
|  | AFUB_075680 (GliZ)_3del_R | GGCAGAAATGGACGGCTATC |
|  | AFUB_086150 (FumR/FapR)_5del_F | CGCCAGTGTGCGTCTGACGG |

**Table S2. Gradient protocols used in the HPLC analysis.**

| A solvent  : 20 mM Ammonium formate (pH 5.2) | |
| --- | --- |
| B solvent  : Methanol (>99.9% HPLC grade) | |
| Time (min) | % B |
| 0.00 | 5 |
| 1.00 | 5 |
| 31.00 | 95 |
| 31.00 | 95 |
| 34.00 | 5 |
| 35.00 | 5 |
